# Wastewater-based surveillance of microbial pathogens in GCC countries (2015–2025): a scoping review and questionnaire survey with stakeholders

**DOI:** 10.3389/fpubh.2026.1786753

**Published:** 2026-04-22

**Authors:** Bushra Alghamdi, Norah Albedah, Turki Almalki, Sami Almudarra, Pasi Penttinen, Changzhi Wang, Pei-Ying Hong

**Affiliations:** 1 Gulf Center for Disease Prevention and Control, Gulf Health Council, Riyadh, Saudi Arabia; 2 Center of Excellence on Smart Health, King Abdullah University of Science and Technology (KAUST), Thuwal, Saudi Arabia

**Keywords:** antimicrobial resistance, omics sequencing, public health, quantitative PCR, viruses, wastewater-based epidemiology

## Abstract

**Background:**

Wastewater-based surveillance (WBS) of microbial pathogens has become an increasingly useful approach to monitor public health at the population level. However, these efforts varied widely in scope, methodology and focus within the GCC region. It remains unclear how WBS is being used across the region, and to what extent it can inform decision-making or contribute to long-term surveillance infrastructure within the GCC. This review aimed to critically assess WBS studies conducted across GCC and to provide perspective on how to further strengthen the ability of WBS to inform and provide early detection on the emergence of health concerns arising from microbial contaminants that are circulating in the community.

**Methods:**

This review was conducted following the PRISMA extension for scoping reviews guidance, aiming to identify and critically evaluate peer-reviewed studies that applied WBS across GCC between January 2015 and October 2025. A structured English-language literature search was carried out on the Web of Science, Scopus, and Google Scholar. Search results were screened against predefined inclusion and exclusion criteria. A survey was conducted with GCC stakeholders involved in the execution of wastewater surveillance, and their responses were studied to align actual WBS activities against that reported in the literature.

**Results:**

A total of 26 studies met the inclusion criteria for this review, with uneven distribution of studies published across the GCC countries (*n* = 6). The main targets reported in the WBS studies are antimicrobial resistance (AMR) and SARS-CoV-2. Majority of the studies report qualitative presence of microbial targets and lack quantitative measurements that are required to facilitate decision-making and intervention measures. Emerging methods and technology that can enable WBS were discussed to facilitate future WBS effort in GCC.

**Conclusion:**

Although WBS holds significant promises to enhance public health surveillance in the GCC, its potential remains underutilized. Moving forward, addressing capacity training and providing sustainable long-term funding mechanisms, standardizing methodological differences and/or providing a guideline that detail the best practices, promoting a consortium-based surveillance system and initiating research that can facilitate the utilization of WBS data to inform decision-making processes would be crucial for the successful integration of WBS into the region's public health framework.

## Background

Wastewater-based surveillance (WBS) has become an increasingly useful approach to monitor public health at the population level (1–4). Its role became more visible during the COVID-19 pandemic. However, WBS is not restricted to surveying only COVID-19 and its value go beyond monitoring only a single pathogen. By detecting various types of biological (e.g., antimicrobial resistance genes (5, 6), enteric viruses (6, 7) and bacterial pathogens) (8) and chemical (e.g., illicit drugs, pharmaceutical compounds etc.) (3, 9) contaminants shed via human waste, WBS can provide much information about the health status of the community contributing to the wastewater. Examples of such information include ongoing or early indication of infectious disease trends (6), the spread of antimicrobial resistance (AMR) (6) and/or other emerging risks (7) within the community. WBS offers a non-invasive approach that does not reveal confidential, sensitive information at the individual level. Hence, compared to clinical surveillance, where bioethics approval is needed, WBS offers a potentially rapid and cost-effective approach to shed insights on public health.

In the Gulf Cooperation Council (GCC) region, interest in WBS has grown over recent years. Shared pressures such as rapid urban development and population growth, increasing needs for water reuse, and transboundary health risks make coordinated surveillance efforts especially relevant (10, 11). Several studies from the region, including Saudi Arabia (10–20), Qatar (21–26), the United Arab Emirates (UAE) (27–31), Kuwait (32–34), and Bahrain (35) have explored WBS applications for SARS-CoV-2, AMR markers and human enteric viruses (Supplementary Table S1 for the full list of literature). However, these efforts varied widely in scope, methodology and focus within the GCC region, and in comparison, to that of other non-GCC countries. As a result, it remains unclear how WBS is being used across the region, and to what extent it can inform decision-making or contribute to long-term surveillance infrastructure within the GCC.

In this review, we aimed to critically assess WBS studies that focused on biological markers, and were conducted across GCC countries over the duration of 2015 through 2025. By compiling and comparing information on genetic targets, detection methodology and sampling strategies, we examined how WBS has been used in different GCC countries and what role it currently plays in public health monitoring. We further identify key gaps in design and implementation. We also provide perspective on the use of new technologies to further strengthen the ability of WBS to inform and provide early detection of the emergence of health concerns arising from microbial contaminants that are circulating in the community.

## Methodology

### Literature collection, screening and analysis

This review was conducted following the PRISMA extension for Scoping reviews (36), aiming to identify and critically evaluate peer-reviewed studies that applied WBS across GCC countries between 2015 and 2025. We limited the search to the past 10 years as majority of the wastewater-based surveillance effort on biological markers happened during and after COVID (2019). In the GCC, majority of the countries still do not have the trained capacity and existing infrastructure to perform wastewater-based surveillance effort on biological markers prior to COVID-19. A structured literature search was carried out on the Web of Science, Scopus, PubMed and Google Scholar. To avoid biasing the scope toward specific pathogens or detection targets, no additional filters were applied beyond geographic relevance. The search was restricted to articles published from January 2015 to October 2025. The literature search utilized keywords related to wastewater surveillance (including “wastewater-based surveillance,” “wastewater monitoring,” “sewage monitoring,” and “wastewater-based epidemiology”) in combination with geographic identifiers for the target region (including “Gulf Cooperation Council,” “GCC,” “Saudi Arabia,” “United Arab Emirates,” “UAE,” “Kuwait,” “Qatar,” “Oman,” and “Bahrain”). The exact Boolean search query used across all four databases is provided in the Data sheet 1.

**Figure 1:**
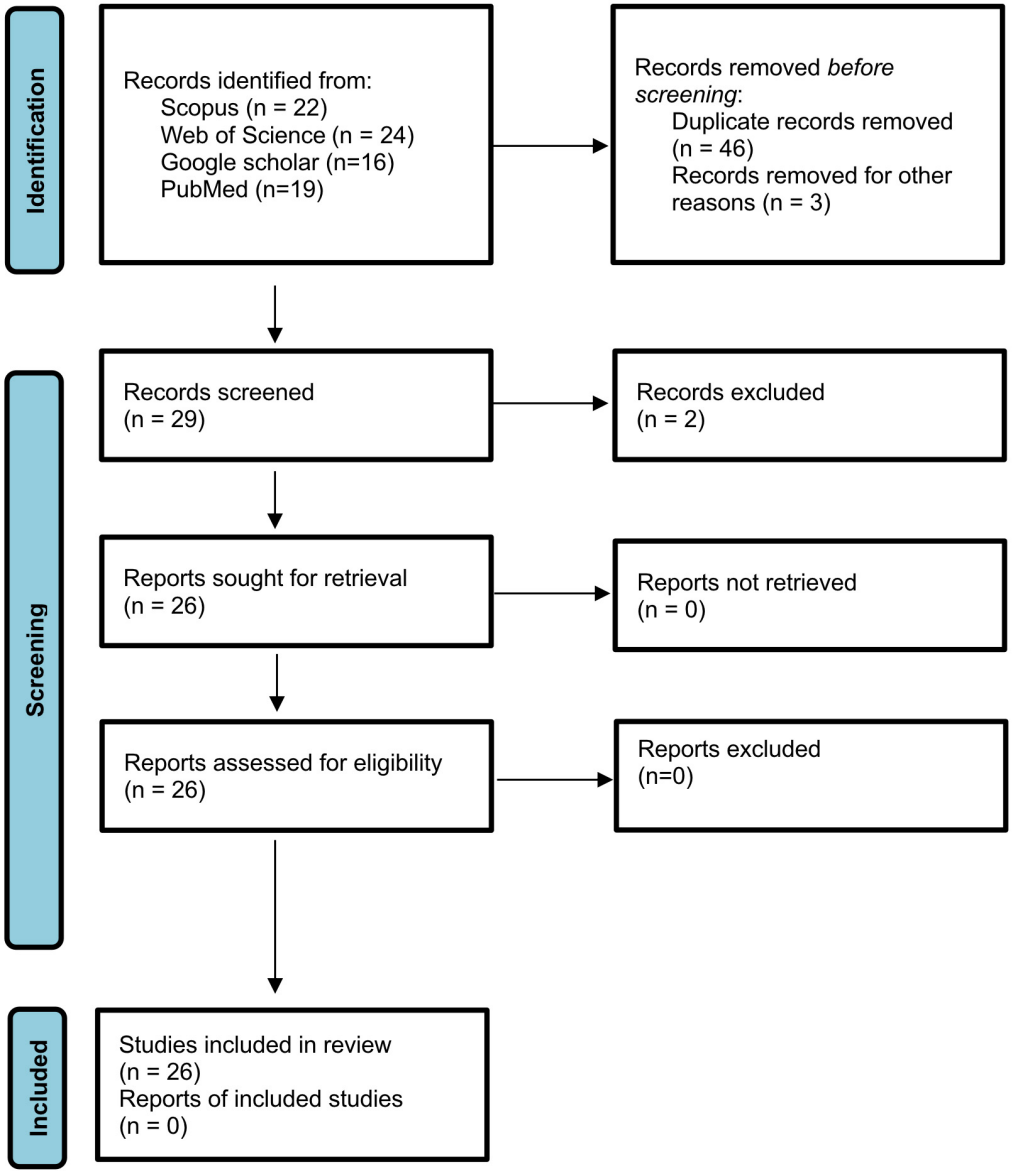
PRISMA 2020 flow diagram for systematic reviews.

Studies were included based on the following criteria: (1) peer-reviewed original research articles; (2) conducted in at least one GCC country; (3) involving direct sampling and analysis of wastewater; and (4) reporting experimental results using molecular, genomic, or culture-based approaches. Exclusion criteria encompassed (1) review papers, editorials, reports, or conference abstracts and (2) modeling or clinical studies lacking environmental samples. For each eligible study, metadata were extracted including the country, year of publication, wastewater type (e.g., municipal, hospital, marine), genetic target(s), detection method (e.g., RT-qPCR, metagenomics, culture-based isolation), sample size, and sampling timeframe. The number of literatures retrieved at each stage of the search process is detailed in Figure 1. Finally, the list of literature and the associated information is compiled into a comparative summary table to support cross-study synthesis (Supplementary Table S1).

### Survey

A questionnaire was prepared with questions devised to understand the status of WBS in the GCC, as well as the current and future priority goals for WBS in the GCC (Data sheet 2). The survey was conducted by inviting targeted stakeholders to participate in answering the questionnaires (demographics shown in Supplementary Table S2). In total, 13 responses were obtained, with two responses from Bahrain, four from Qatar, two each from UAE, Oman and Kuwait, and one response from Saudi Arabia. Ten of the respondents represent the Ministry of Health from their respective countries while the other three respondents are from academic/research institutions. Stakeholders with expertise in wastewater surveillance were identified through consultation with representatives from the Gulf CDC Permanent Communication Network and the Laboratory Technical Working Group. The identified stakeholders were then invited to participate in the survey via email or phone call.

## Results

### Findings from scoping review

#### Geographic distribution and the scale of WBS

**Figure 2:**
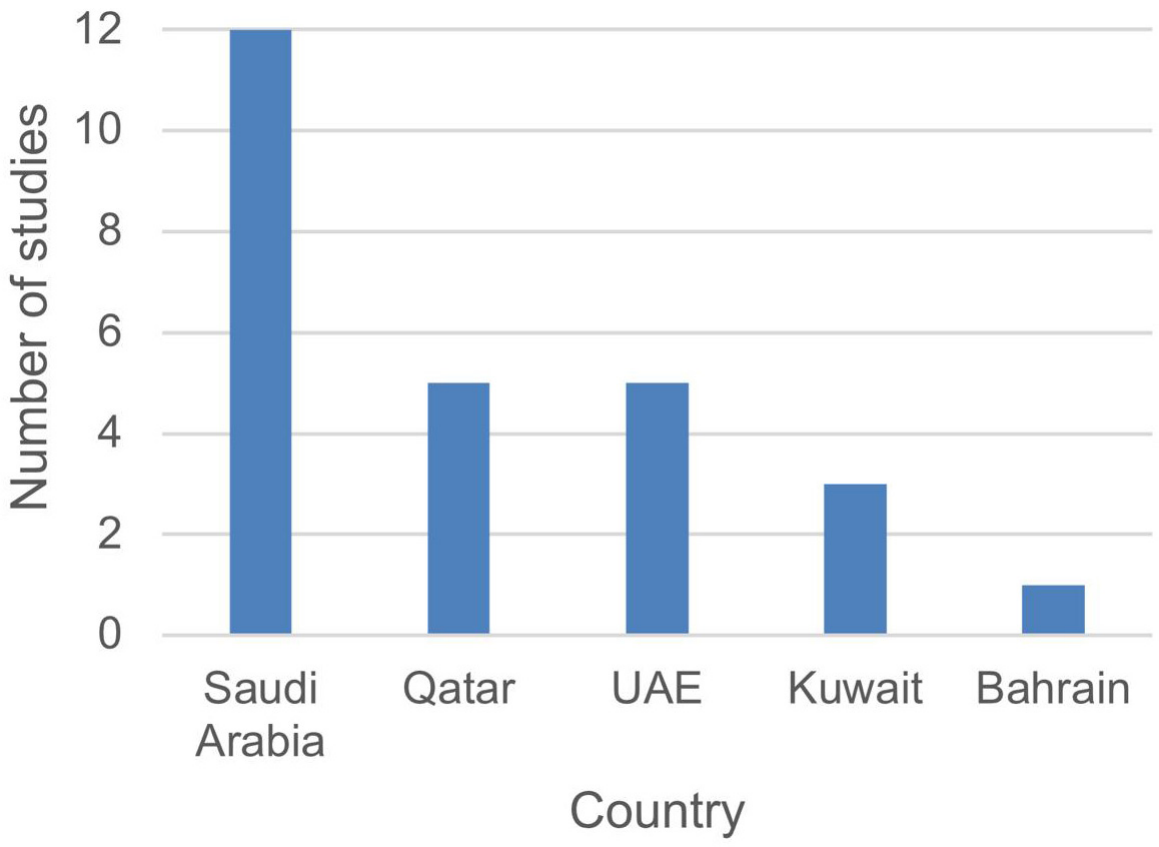
Peer-reviewed WBS studies from GCC countries (2015–2025, *n* = 26).

This review identified 26 peer-reviewed studies published between 2015 and 2025 that reported WBS studies across five GCC countries. Saudi Arabia accounted for half of the reviewed studies (*n* = 12), followed by Qatar (*n* = 5) and the United Arab Emirates (*n* = 5), Kuwait (*n* = 3), and Bahrain (*n* = 1; Figure 2). Oman yielded no peer-reviewed publications fitting the inclusion criteria during the review period. 85% of the published studies are open access which facilitates the access of readers interested in the topic.

**Figure 3:**
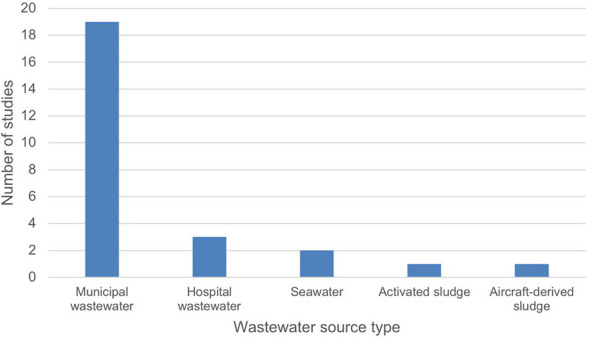
Wastewater source type of peer-reviewed WBS studies from GCC countries (2015–2025, *n* = 26).

A broad range of wastewater or wastewater-contaminated sample types were covered in the reviewed studies (Supplementary Table S1). While municipal wastewater remained the primary source of samples (*n* = 19), it was noted that not all studies (*n* = 6) included information on the equivalent population size captured by each sampling point of the wastewater treatment time. From our survey and interview with the relevant stakeholders, majority of them have noted that there is insufficient knowledge on the wastewater network that is served by each of the studied wastewater treatment plants to allow traceback efforts, if pathogens of interest are detected in high abundance. If the objective of wastewater-based surveillance is to protect public health, the inability to perform traceback efforts would limit one to perform epidemiological tracing or to implement appropriate intervention measures. Besides municipal wastewater, several studies extended surveillance to include hospital wastewater (*n* = 3), seawater impacted by treated discharge (*n* = 2), activated sludge (*n* = 1), and aircraft-derived wastewater (*n* = 1; Figure 3). While performing WBS from hospitals would provide significant insights into the diversity of pathogens that are causing infections in the hospital environment, these insights derived from hospital wastewaters may not suffice if the purpose of WBS is for early monitoring of threats that are circulating in the local community prior to causing infection or illnesses. However, wastewater surveillance on hospital wastewater may potentially reveal insights on how antibiotic prescribing practices and hospital antimicrobial stewardship can influence environmental antibiotic loads and resistance gene dissemination into the non-nosocomial environment (12, 16, 37), including horizontal gene transfer and contamination of surface water systems, which is especially concerning in places where sanitation infrastructure is lacking.

All published literature was conducted within a single country, with small sample sizes (typically fewer than 100), and conducted over a short surveillance duration that often lasted less than 1 year.

#### Investigated types of samples, target organisms and genetic markers

The reviewed studies primarily focused on two types of biological targets: antibiotic resistance genes (ARG) associated with antibiotic-resistant bacteria, and pathogenic viruses, most notably SARS-CoV-2 during the COVID-19 pandemic. A handful of studies also monitored human enteric viruses such as adenovirus, norovirus, and hepatitis A and E. There is an observable trend between the type of surveyed target and citation numbers (Supplementary Table S1) Specifically, from the citation numbers, the ones that were generally of higher interest were the ones related to SARS-CoV-2 during the pandemic phase. This is followed by AMR and then enteric viruses at different phases, before and after the pandemic.

Majority of the studies focused on reporting the qualitative presence of the biological targets, which are reported in units such as percentage of samples positive with the target (i.e., occurrence percentage), as threshold cycle value when using quantitative PCR or as percentage relative abundance when using omics-based sequencing approaches (Supplementary Table S1). Only a few studies conducted in GCC provided quantitative abundances of the targets in the form of copy numbers or log CFU per volume of wastewater (Supplementary Table S1).

#### Analytical approaches

As shown in Supplementary Table S1, the most common analytical method used in most studies (*n* = 17) conducted in GCC is quantitative PCR (qPCR), which includes reverse transcription-qPCR when used to detect RNA viruses like SARS-CoV-2. To detect SARS-CoV-2 and enteric viruses, the first step is to concentrate these viruses, and the commonly used approach includes either polyethylene glycol to precipitate viruses, flocculation with beef extract or filtration through membrane. Each of these methods are known to have differences in recovery efficiency (38), especially when used on wastewater matrices that have wide variability in turbidity and organic matter concentrations and can affect recovery of viruses. Similarly, differences in the qPCR protocols (e.g., different types of standard templates, thermal cycling protocol etc.) and the nucleic extraction methods can also result in differences in quantitative results. Among the published GCC studies, most do not report on the recovery of spiked internal standards. Nor do they report the qPCR amplification efficiencies in accordance with the Minimum Information for Publication of quantitative real-time PCR experiments (MIQE) guideline (39). Hence, cross-studies comparisons among the published GCC literature cannot be easily conducted. There is a need to have common standard operating protocols, standardized analysis and reporting protocols in order to achieve comparability.

To detect AMR, culture-based approaches are often used in most of the GCC studies. This involves filtering the diluted wastewater samples through membranes to retain the biomass, and the membrane with the retained biomass is then placed on agar media plates for incubation. The cultivation substrata used in most studies include selective media that preferentially favor the growth of certain types of bacteria (e.g., *E. coli* or Enterobacteriaceae) and/or are supplemented with antibiotics (e.g., meropenem or ceftazidime to select for the growth of colonies that most likely exhibit traits for extended spectrum beta-lactamases). Majority of the studies implemented an appropriate QC step, which is to perform PCR and sequencing on the relevant genes—for example, 16S rRNA genes to determine the phylogenetic identity or antibiotic resistance genes that confer the observed resistance. In instances where no confirmation experiments on the identities and functional traits of the isolates are performed, these studies overlook the possibility of false-positives and may overestimate the actual prevalence of the AMR issue.

### Findings from stakeholder's survey

#### Surveyed types of samples, target organisms and genetic markers

**Figure 4:**
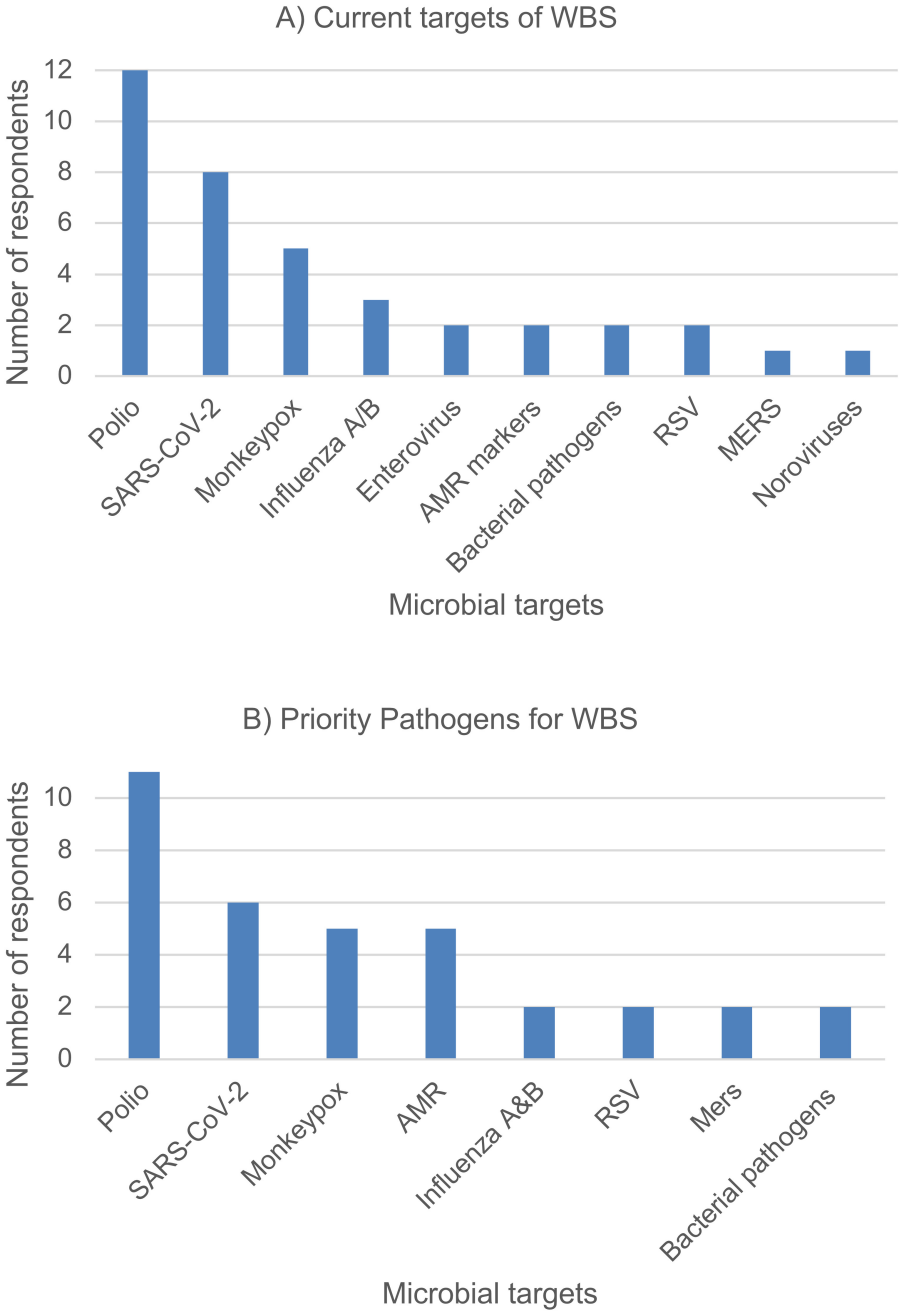
A list of microbial targets that are **(A)** currently monitored, compared against **(B)** the perceived priority targets for WBS as mentioned by surveyed GCC stakeholders.

From our survey response, majority of the stakeholders are currently monitoring infectious viruses such as COVID-19, poliovirus, Influenza A/B and Mpox, all of which also impose a risk of public health outbreak and with high disease burden (Figure 4A). Other targets such as AMR, respiratory syncytial virus (RSV), enterovirus, Norovirus, Middle East Respiratory Syndrome Coronavirus (MERS-CoV) and bacterial pathogens were also monitored by WBS (Figure 4A). However, when cross-referenced against the stakeholders' priority target list, which was a list of biological markers collated from our survey done with the relevant stakeholders (Figure 4B), it was noted that there was a mismatch in terms of AMR being listed high in the priority target list but not as widely monitored by most of our surveyed stakeholders in their current WBS effort. It was also observed that the listed targets that are high on stakeholders' priority list are often not the ones that are reported in published literature, likely because of the sensitivity associated with positive detection of these targets. This is aligned with the survey responses in which most of the information gathered by the stakeholders is disseminated internally through reports to the local health units or environmental public health institutions and not shared widely with the public. Alternatively, a mismatch between the stakeholders (ministries) and the authors of the studies (probably mainly research) may be due to government and academia not closely communicating with each other on which biomarkers to focus monitoring effort on.

#### Emerging technologies that can be implemented for Gulf WBS effort

As part of the response that we got from our survey questionnaire and from a subsequent Gulf CDC workshop that was conducted with the relevant stakeholders, there is a significant interest in emerging technologies to improve the ability and scope of WBS. In this review, we provide examples of emerging technologies that can be implemented to aid Gulf WBS effort.

##### High-throughput PCR platforms

Given the wide variety of contaminants that can be monitored by WBS (Supplementary Table S3), high-throughput PCR platforms can facilitate simultaneous detection. Such platforms utilize microfluidic dynamic arrays instead of the well plate format, but users still prepare their PCR reaction mixture as per usual before loading them to inlet wells on the dynamic arrays and then carry out the thermal cycling program. The current high throughput dynamic arrays are in the form of 96 by 96 format, which allows 9,216 individual data points to be obtained per array. Such kinds of microfluidic arrays only require nanoliters of each reaction mixture and are therefore particularly suitable to quantify a wide diversity of gene targets in samples with low DNA yield. However, one limitation of such platform is that the primers would have to be designed to have similar optimal annealing temperatures since all reactions run simultaneously under the same thermal cycling conditions. Wang et al. (40) demonstrated the use of such array to simultaneously quantify the abundances of 285 ARGs, nine transposase genes and 16S rRNA genes in soils irrigated with treated wastewater. In the study, the authors only performed delta threshold cycle (ΔCt) to a reference gene and did a fold-change comparison with no mention on the absolute abundance of gene copies. The majority of GCC studies that utilized high-throughput PCR platforms also adopt this analysis and thus only provide qualitative determination of the target and the trendline changes of the target over the study period.

Ideally, quantitative microbial risk assessment which can aid in assessing risks derived from infectious biomarkers would benefit relatively more from quantitative abundance measurements than qualitative measurement. Alternatively, quantitative abundance measurements can also facilitate comparison of the extent of microbial contaminants in wastewater across the GCC countries and to assess removal efficacy of treatment technologies. For such quantitative purposes, Zhao et al. (41) also developed a high-throughput qPCR array to quantitatively profile 19 genes related to arsenic cycling in environmental samples. This is reliant on standard curve method of quantification of 16S rRNA genes that were also imprinted on the same high-throughput qPCR platform, and the subsequent normalization of copies of each gene against the copies of 16S rRNA genes. To further utilize such quantitative assessment for risk determination, one can possibly also imprint a single-copy housekeeping gene of bacteria (e.g., gyrB or rpoA) so that a normalization of the target gene against these housekeeping gene would provide an estimation of the target cell numbers.

##### Omics sequencing

Among the current list of published papers from GCC, only a small subset (*n* = 3) employed metagenomics to enable untargeted profiling of microbial communities and resistomes. Metagenomics, being DNA-based, can only provide information on who is there (i.e., taxonomic and phylogenetic information) and what is there (i.e., functional genes diversity). An earlier review paper by (42) opined the use of metagenomics as a tool to monitor reclaimed water quality. Likewise, metagenomics can serve in WBS to address questions such as the type of biological gene diversity and their relative abundances in a water sample (14, 43). The earlier review by Hong et al. provided a summary of the different sequencing platforms and bioinformatic tools that are available to analyze the sequences, and readers of this report are encouraged to refer to that earlier paper. However, a key recent advance which was not reflected in the earlier review paper is the availability of metagenome sequins. Sequins are a set of synthetic DNA controls that reflect the sequence complexity, GC content, phylogenetic diversity and abundance of a natural microbial community. By spiking in known amount of these sequins to the sample prior to preparing the sample for metagenomic, these synthetic sequences can serve as internal controls and can aid in providing a quantitative estimate of abundances of sequenced targets. Recent demonstrations of sequins for quantitative metagenomics of bacteria and viruses can be found here (44, 45), and although the use of quantitative metagenomics is not widely adopted yet, further testing on the use of sequins to enable quantitative metagenomics in WBS should be considered by GCC stakeholders.

##### Tools to enable prediction or forecasting

**Figure 5:**
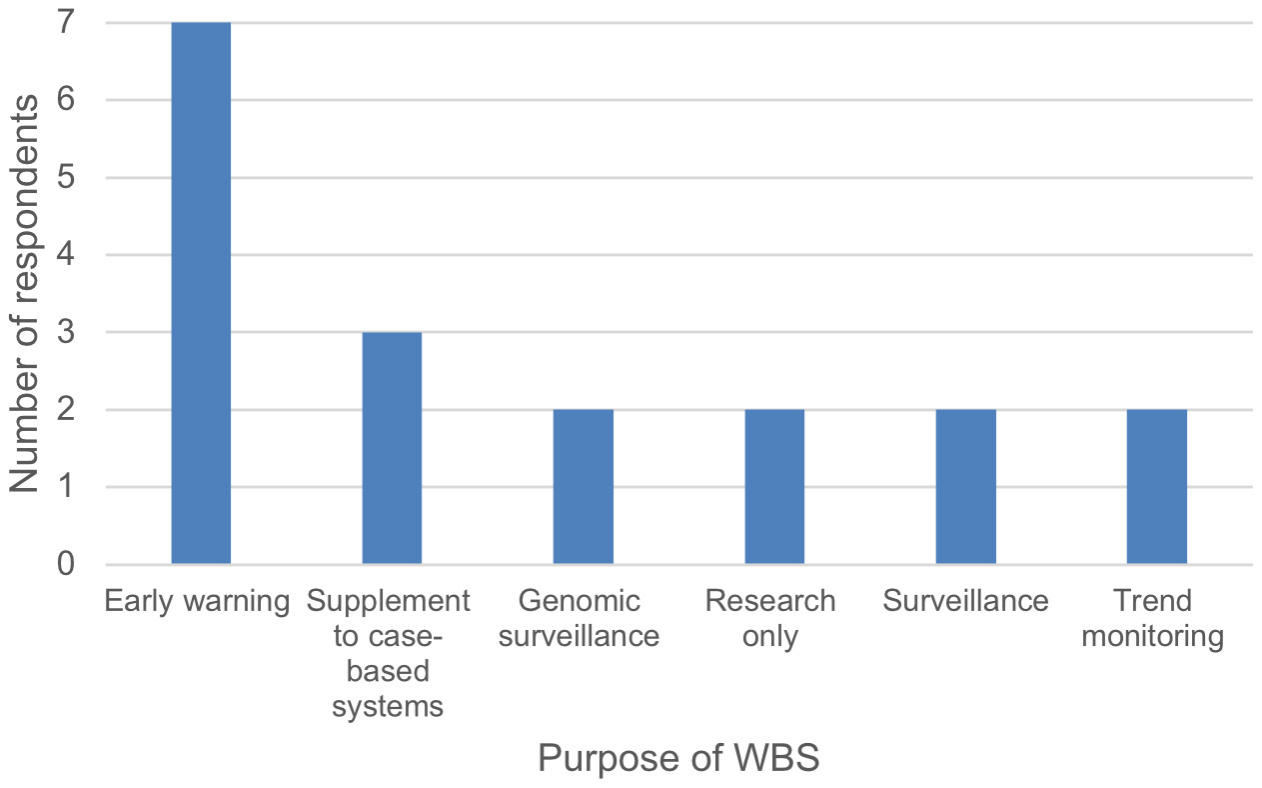
Surveyed responses indicating what GCC stakeholders would like to seek from WBS.

In our survey with the GCC stakeholders, many have expressed concerns that integrating the data derived from WBS to inform action plans and decision-making has been poor. There is a lack of knowledge on how to utilize these data to inform action plans and decisions. They have also highlighted the need for WBS-derived data to aid in forecasting the occurrence of public health concerns that may arise from the positive detection of certain key pathogens (Figure 5).

In the GCC, the closest matched tool that has been demonstrated to enable prediction or forecasting is soft sensors (i.e., sensors that are built based on machine learning approach), whereby chemical and physical parameters (e.g., COD, turbidity, pH etc) of wastewater that were used to estimate total bacteria and virus cell density in the water (46–49). The current soft sensors would serve to monitor for aberrations in water quality, the performance of wastewater treatment facilities, detect leaks or intrusions in distribution systems. However, more research needs to be done to develop a soft sensor that can predict for the occurrence of pathogen and their abundance. This can be done by first identifying the key unique marker that is associated with the pathogen, then subsequently collecting data that maps the abundance of that unique marker with that of the abundance of pathogen in a large sample dataset and then training models using these datasets to predict and forecast future events. While such tools are valuable and can aid in the decision-making process among stakeholders, these tools remain in their infancy and require more research and development prior to application.

## Discussion

Outside the GCC, WBS is increasingly being used as a practical tool to complement clinical reporting, especially for tracking pathogens and antimicrobial resistance at community scale. For example, large, multi-country wastewater metagenomics studies show that resistome profiles differ in consistent, region-specific ways, and that looking at gene context levels can help separate locally circulating signals from patterns that suggest wider spread of these genetic elements (50). Recent perspectives also describe how programs are moving toward standardized sampling and reporting (e.g., clear catchment definitions, population and flow metadata, and consistent sampling frequency) so that trends can be compared across sites and over time, with uncertainty reported in a way public-health teams can actually use (51). Finally, implementation work stresses that scaling up depends as much on governance as that on lab capacity: namely, clear data ownership, privacy safeguards, and transparent communication with communities (52). Together, these efforts offer concrete practices that the GCC can adapt when building WBS beyond pilot studies.

Although WBS holds significant promises to enhance public health surveillance in the GCC, its potential remains underutilized. WBS in the GCC region faces several key challenges. First, the number of published peer-reviewed studies remains low, with uneven distribution across countries and a complete absence from Oman. This distribution suggests that WBS activity is concentrated in a few countries within GCC, although based on survey results, all participants noted that WBS is already in place or to be initiated soon in their respective countries. This uneven distribution in published WBS literature also suggests a possible lack of sufficient funding, trained human capacity and other support mechanisms to facilitate WBS within the GCC region. Notably, as most of the WBS published in literature are conducted only within a specific country with no demonstrations of cross-country efforts, this indicates both the exploratory nature of WBS in the region and the need for more coordinated, large-scale efforts within the GCC. This is particularly important as dissemination of microbial pathogens is not restricted to within a particular country's borders and global/regional travelers have been shown previously to introduce new microbial-associated contaminants to the local environment (53).

The second challenge noted in this scoping review is that there is large methodological variability such as differences in sample types, detection methods, and reporting standards across the literature published by GCC researchers. These differences hinder meaningful cross-country comparisons and data sharing. In addition, the lack of this information does not allow the implementation of quality appraisal tools (e.g., Joanna Briggs Institute Environmental Health Quality Assessment Framework) when assessing the methodology quality and/or risk of bias of studies included in this scoping review. Furthermore, the lack of quantitative measurements in most studies impedes the ability to infer risks and dissemination extent of the detected targets, which are insights needed for better decision-making to protect public health. This is made obvious in which most studies lack integration with clinical or epidemiological data, reducing their relevance for real-time public health action and regional coordination. To achieve such goal, in addition to the need for quantitative estimates of the pathogens, there will also be a need for other types of meta-data, for example, the growth rates or infectious rates of the pathogen within the population after a certain exposure duration. Such information is not easily obtained by WBS alone unless a more concerted effort to collect all related meta-data can be put in place. However, once such information is available, one can develop epidemiological-based models to map the extent of dissemination of a pathogen that is detected in the wastewater. To the best of our knowledge, such demonstrations have not been reported yet in the GCC.

Moving forward, addressing capacity training and providing sustainable long-term funding mechanisms, standardizing methodological differences and/or providing a guideline that detail the best practices, promoting a consortium-based surveillance system and initiating research that can facilitate the utilization of WBS data to inform decision-making processes would be crucial for the successful integration of WBS into the region's public health framework. With better insights obtained from WBS, clearer recommendations regarding the types of sanitation infrastructure (e.g., advanced wastewater treatment technologies like membrane filtration, advanced oxidation processes etc.) can be implemented to further mitigate environmental dissemination of microbial contaminants and antimicrobial resistance threats. Such efforts would in turn further protect the region's environmental and community health.

## Data Availability

The original contributions presented in the study are included in the article/Supplementary material, further inquiries can be directed to the corresponding author.
