# Supplementary material for: Wastewater-based surveillance of microbial pathogens in GCC countries (2015–2025): a scoping review and questionnaire survey with stakeholders
Source: Front Public Health. 2026 Apr 22;14:1786753. doi: 10.3389/fpubh.2026.1786753 (PMC13144031; doi:10.3389/fpubh.2026.1786753)
Supplement: Supplementary file 3 [file Table_3.docx]

**Appendix 3.** Primers and probes reported in literature to target various types of viral, bacterial and protozoal pathogens.

| **Pathogen types** | **Gene target** | **Primer and/or probe sequences** | **Amplicon size** | **Reporter type** | **Limit of detection** | **Amplification efficiency (%)** | **Ref** |
| --- | --- | --- | --- | --- | --- | --- | --- |
| **Viral** |  |  |  |  |  |  |  |
| Norovirus | GI (ORF1-ORF2 junction) | \| Forward: CGYTGGATGCGNTTYCATGA \| \| --- \| \| Reverse: CTTAGACGCCATCATCATTYAC \| \| Mixed Probes (a): FAM-AGATYGCGATCYCCTGTCCA-TAMRA \| \| Mixed Probes (b): FAM-AGATCGCGGTCTCCTGTCCA-TAMRA \| | 86 | Taqman | 10 copies/PCR reaction | 98.0 | (Kageyama, Kojima et al. 2003) |
|  | GII (ORF1-ORF2 junction) | \| Forward: CARGARBCNATGTTYAGRTGGATGAG \| \| --- \| \| Reverse: TCGACGCCATCTTCATTCACA \| \| Probe: FAM-TGGGAGGGCGATCGCAATCT-TAMRA \| | 98 | Taqman |  | 104.4 |  |
| Hepatitis | 5’ non-coding region | \| Forward: TCACCGCCGTTTGCCTAG \| \| --- \| \| Reverse: GGAGAGCCCTGGAAGAAAG \| \| Probe: FAM-CCTGAACCTGCAGGAATTAA-MGBNFQ \| | 157-188 | Taqman | 0.05 infectious particle/PCR reaction | 106.3 | (Costafreda, Bosch and Pinto 2006) |
| Rotavirus | Non-structural protein 3 | \| Forward: ACCATCTWCACRTRACCCTC \| \| --- \| \| Reverse: GGTCACATAACGCCCCTATA \| \| Probe: FAM-ATGAGCACAATAGTT/BHQ1/AAAAGCTAACACTGTCAA \| | 87 | Taqman | 44 genome copies/PCR reaction | 99.7 | (Freeman, Kerin et al. 2008) |
| Adenovirus | Hexon gene | \| Forward: GGACGCCTCGGAGTACCTGAG \| \| --- \| \| Reverse: ACIGTGGGGTTTCTGAACTTGTT \| \| Probe: FAM-CTGGTGCAGTTCGCCCGTGCCA-BHQ \| | 96 | Taqman | 5 genome copies (AdV40), 8 genome copies (AdV41), 350 genome copies (AdV3) /PCR reaction | 92.5 | (Jothikumar, Cromeans et al. 2005) |
|  | Penton base | \| Forward: CGTCTTCAAYCGCTT \| \| --- \| \| Reverse: TGTAGACGTAGGGACAGG \| \| Donor Probe 1: CCGTCAGTGAAAACGTGC-Fluorescein \| \| Acceptor Probe 2: RED640-GCTCTCACAGATCACGGG-P \| | 185 | FRET | 0.1-1000 tissue culture infective dose (TCID50)/mL | Not reported | (Buckwalter, Teo et al. 2012) |
| Enterovirus |  | \| Forward: CCTCCGGCCCCTGAATG \| \| --- \| \| Reverse: ACCGGATGGCCAATCCAA \| \| Probe: FAM-CGGAACCGACTACTTTGGGTGTCCGT-TAMRA \| | 113 | Taqman | 2 genome equivalents/L | 90.0 | (Cashdollar, Brinkman et al. 2013) |
| **Bacterial** |  |  |  |  |  |  |  |
| *Legionella pneumophila* | Mip (macrophage infectivity potentiator surface protein) | \| Forward: GCAATGTCAACAGCAA \| \| --- \| \| Reverse: CATAGCGTCTTGCATG \| \| Probe 1: CAACTTATCCTTGTCTGTAGCT-FAM \| \| Probe 2: RED640-TGATGTGGCATCGGTTG-P \| | 159 | FRET | 10 organisms/PCR reaction | Not reported | (Wilson, Yen-Lieberman et al. 2003) |
|  |  | \| Forward: TTCATTTGYTGYTCGGTTAAAGC \| \| --- \| \| Reverse: AWTGGCTAAAGGCATGCAAGAC \| \| Probe: FAM-AGCGCCACTCATAG-MGB \| | 66 | Taqman | 60 genome copies/L | 97.6 | (Behets, Declerck et al. 2007) |
|  | dotA (required to create organelle inside eukaryotic host) | \| Forward: ATTGTCTCGCGCGATTGC \| \| --- \| \| Reverse: CCGGATCATTATTAACCATCACC \| \| Probe: FAM-ATACAGCAAATGTATGTGACTT-MGB \| | 80 | Taqman | 7 copies/PCR reaction | 113.8 | (Yanez, Carrasco-Serrano et al. 2005) |
|  | 23S-5S rRNA spacer region | \| Forward: GTACTAATTGGCTGATTGTCTTGACC \| \| --- \| \| Reverse: CCTGGCGATGACCTACTTTCG \| \| Probe: CalOrg-ATCGTGTAAACTCTGACTCTTTACCAAACCTGTGG-BHQ \| | 270 | Taqman | 3 genome copies/PCR reaction | 102.6 | (Yang, Benson et al. 2010) |
| *E. coli* O157:H7 | Z3276 (putative fimbrial protein) | \| Forward: GCACTAAAAGCTTGGAGCAGTTC \| \| --- \| \| Reverse: AACAATGGGTCAGCGGTAAGGCTA \| \| Probe: FAM-CGTTGGCGAGGACC-MGBNFQ \| | 130 | Taqman | 8 CFU/PCR reaction | 97.8 | (Li and Chen 2012) |
|  | ybiX (Fe(II)-dependent oxygenase) | \| Forward: CGCCATGCTGTTTGAACTGG \| \| --- \| \| Reverse: CAGGATCTCTTCATTTTCAC \| \| Probe: FAM-ATTCAGAATATTCAGTCGCTGAAAAGC-BHQ-1 \| | 82 | Taqman | 100 genome copies/PCR reaction | 100.5 | (Wong, Paschos et al. 2014) |
|  | tir (translocated intimin receptor) | \| Forward: GTCAGCTCATTAACTCTACGGG \| \| --- \| \| Reverse: GCCTGTTAAGAGTATCGAGCG \| | 207 | Sybr Green | 10 cells/mL | 93.3 | (Clark, Gilbride et al. 2011) |
|  | stx-1 (Shiga toxin 1) | \| Forward: GACTGCAAAGACGTATGTAGATTCG \| \| --- \| \| Reverse: ATCTATCCCTCTGACATCAACTGC \| \| Probe: FAM-TGAATGTCATTCGCTCTG-MGBNFQ \| | 150 | Taqman | 100 copies/PCR reaction | 100.9 | (Sen, J et al. 2011) |
|  | stx-2 (Shiga toxin 2) | \| Forward: ATTAACCACACCCCACCG \| \| --- \| \| Reverse: GTCATGGAAACCGTTGTCAC \| \| Probe: VIC-CAGTTATTTTGCTGTGGATATA-MGBNFQ \| | 200 | Taqman | 100 copies/PCR reaction | 100.9 |  |
|  | eae (*E. coli* O157:H7-specific intimin) | \| Forward: GTAAGTTACACTATAAAAGCACCGTCG \| \| --- \| \| Reverse: TCTGTGTGGATGGTAATAAATTTTTG \| \| Probe: NED-AAATGGACATAGCATCAGCATA-MGBNFQ \| | 106 | Taqman | 100 copies/PCR reaction | 98.8 |  |
| *Campylobacter jejuni* | VS1 (fragment of unknown function) | \| Forward: GAATGAAATTTTAGAATGGGG \| \| --- \| \| Reverse: GATATGTATGATTTTATCCTGC \| \| Probe: FAM-TTTAACTTGGCTAAAGGCTAAGGCT-TAMRA \| | 358 | Taqman | 6-15 CFU/PCR reaction | 98.8 | (Yang, Jiang et al. 2003) |
|  |  | \| Forward: GAATGAAATTTTAGAATGGGG \| \| --- \| \| Reverse: GATATGTATGATTTTATCCTGC \| | 358 | Sybr Green | 200 CFU/mL | 90 |  |
|  | Putative gluconate 2-dehydrogenase subunit | \| Forward: CTGAATTTGATACCTTAAGTGCAGC \| \| --- \| \| Reverse: AGGCACGCCTAAACCTATAGCT \| \| Probe: TCTCCTTGCTCATCTTTAGGATAAATTCTTTCACA \| | 86 | Taqman | 1 CFU/PCR reaction | 98.8 | (Nogva, Bergh et al. 2000) |
|  | hsp60 (heat shock protein) | \| Forward: CAAGTTGCTACAATCTCAGCCA \| \| --- \| \| Reverse: GATAACACCATCTTTGCCCACT \| | 90 | Sybr Green | 53 CFU/mL | 91.2 | (Park, Hanning et al. 2011) |
| *Shigella* spp. | ipaH (Type III effector) | \| Forward: ACCATGCTCGCAGAGAAACT \| \| --- \| \| Reverse: TACGCTTCAGTACAGCATGC \| \| Probe: RED610-TGGCGTGTCGGGAGTGACAGC-BHQ \| | 181 | Taqman | 10-150 CFU/L | 95.4 | (Lin, Cheng and Van 2010) |
| *Vibrio cholerae* | ompW (outer membrane protein) | \| Forward: ACATCAGYTTTGAAGTCCTCGC \| \| --- \| \| Reverse: GTGGTGTAATTCAAACCCGC \| | 191 | Sybr Green | 6 copies/PCR reaction | 97.3 | (Rashid, Ferdous et al. 2017) |
|  | ctxA (enterotoxin subunit A) | \| Forward: TTTGTTAGGCACGATGATGGAT \| \| --- \| \| Reverse: ACCAGACAATATAGTTTGACCCACTAAG \| \| Probe: FAM-TGTTTCCACCTCAATTAGTTTGAGAAGTGCCC-BHQ \| | 84 | Taqman | 10 CFU/reaction | 90 | (Blackstone, Nordstrom et al. 2007) |
|  | gbpA (chitin-binding protein) | \| Forward: CCGCAGCTTCCTTCTACAAC \| \| --- \| \| Reverse: GGCTTTGGTTAGCGTCTCAG \| \| Probe: FAM-AACCCAGCAGGTCAAATCATTCCAAGTA-BBQ \| | 206 | Taqman | 100 genome copies/L | 93.1 | (Vezzulli, Stauder et al. 2015) |
| *Yersinia enterocolitica* | ail (attachment and invasion locus) | \| Forward: ATGATAACTGGGGAGTAATAGGTTCG \| \| --- \| \| Reverse: CCCAGTAATCCATAAAGGCTAACATAT \| \| Probe 1: FAM-TGACCAAACTTATTACTGCCATA-MGB \| | 163 | Taqman | 10 CFU/PCR reaction | 90.9 | (Lambertz, Nilsson et al. 2008) |
| *Pseudomonas aeruginosa* | exoT (Type III effector protein) | \| Forward: GGTCTCTATACCAACGGCGA \| \| --- \| \| Reverse: GAACAGGGTGGTTATCGTGC \| | 285 | Sybr Green | 10 cells/mL | 105.3 | (Clark, Gilbride et al. 2011) |
|  | gyrB (DNA gyrase subunit B) | \| Forward: CCTGACCATCCGTCGCCACAAC \| \| --- \| \| Reverse: CGCAGCAGGATGCCGACGCC \| \| Probe: FAM-CCGTGGTGGTAGACCTGTTCCCAGACC-BHQ \| | 220 | Taqman | 730 CFU/mL | Not reported | (Anuj, Whiley et al. 2009, Le Gall, Le Berre et al. 2013) |
|  | ecfX (putative anti-sigma protein) | \| Forward: CGCATGCCTATCAGGCGTT \| \| --- \| \| Reverse: GAACTGCCCAGGTGCTTGC \| \| Probe: YAK-ATGGCGAGTTGCTGCGCTTCCT-BHQ \| | 63 | Taqman |  | Not reported |  |
| *Salmonella enterica* | invA (involved in the invasion of epithelial cells) | \| Forward: TATGCCCGGTAAACAGATGAG \| \| --- \| \| Reverse: GTATAAGTAGACAGAGCGGAGG \| | 252 | Sybr Green | 10 cells/mL | 95.8 | (Clark, Gilbride et al. 2011) |
| *Mycobacterium* spp. | 16S rRNA | \| Forward: CCTGGGAAACTGGGTCTAAT \| \| --- \| \| Reverse: CGCACGCTCACAGTTA \| \| Probe: HEX-TTTCACGAACAACGCGACA(ROX)AACT-P \| | 154 | Taqman | 79 genome copies/PCR reaction | 74.3 | (Garcia-Quintanilla, Gonzalez-Martin et al. 2002, Radomski, Lucas et al. 2010) |
|  | IS6110 (insertion element exclusive to *Mycobacterium tuberculosis* complex) | \| Forward: CTCGACCTGAAAGACGTTATCC \| \| --- \| \| Reverse: CTCGGCTAGTGCATTGTCATA \| \| Probe: FAM-AGTACACAT/ZEN/CGATCCGGTTCAAGCG-IBFQ \| | 141 | Taqman | 10 copies/PCR reaction | 99.9 | (Wang, Lu et al. 2019) |
|  | mpb70 (*Mycobacterium* antigen) | \| Forward: CTCAATCCGCAAGTAAACC \| \| --- \| \| Reverse: TCAGCAGTGACGAATTGG \| \| Probe: FAM-CTCAACAGCGGTCAGTACACGGT-BHQ \| | 133 | Taqman | 10 genome copies/PCR reaction | 102.6 | (Lorente-Leal, Liandris et al. 2019) |
| **Protozoal** |  |  |  |  |  |  |  |
| *Giardia intestinalis* | Small subunit rRNA | \| Forward: GACGGCTCAGGACAACGGTT \| \| --- \| \| Reverse: TTGCCAGCGGTGTCCG \| \| Probe: FAM-CCCGCGGCGGTCCCTGCTAG-TAMRA \| | 62 | Taqman | 0.5 cyst/PCR reaction | Not reported | (Verweij, Schinkel et al. 2003) |
|  | Beta-giardin (structural constituent of cytoskeleton) | \| Forward: CATCCGCGAGGAGGTCAA \| \| --- \| \| Reverse: GCAGCCATGGTGTCGATCT \| \| Probe: FAM-AAGTCCGCCGACAACATGTACCTAACGA-BHQ \| | 74 | Taqman | 1 copy/PCR reaction | 99.6 | (Guy, Payment et al. 2003) |
| *Cryptosporidium parvum* | cowP (wall protein) | \| Forward: CAAATTGATACCGTTTGTCCTTCTG \| \| --- \| \| Reverse: GGCATGTCGATTCTAATTCAGCT \| \| Probe: HEX-TGCCATACATTGTTGTCCTGACAAATTGAAT-BHQ \| | 150 | Taqman | 4 copies/PCR reaction | 90.1 |  |
| *Acanthamoeba* | 18S rRNA | \| Forward: CCCAGATCGTTTACCGTGAA \| \| --- \| \| Reverse: TAAATATTAATGCCCCCAACTATCC \| \| Probe: Cy5-CTGCCACCGAATACATTAGCATGG-BHQ \| | 200 | Sybr Green or Taqman | Sybr Green: 5 cells/PCR reaction  Taqman: 0.8 cells/PCR reaction | 103.1 | (Qvarnstrom, Visvesvara et al. 2006, Fittipaldi, Pino Rodriguez et al. 2011) |
| *Entamoeba histolytica* | Small subunit RNA | \| Forward: ATTGTCGTGGCATCCTAACTCA \| \| --- \| \| Reverse: GCGGACGGCTCATTATAACA \| \| Probe: VIC-TCATTGAATGAATTGGCCATTT-NFQ \| | 72 | Taqman | Not reported | Not reported | (Verweij, Blange et al. 2004) |
|  | Small subunit RNA | \| Forward: AACAGTAATAGTTTCTTTGGTTAGTAAAA \| \| --- \| \| Reverse: CTTAGAATGTCATTTCTCAATTCAT \| \| Probe: ATTAGTACAAACTGGCCAATTCATTCA (no mention of fluorophore and quencher used) \| | 134 | Taqman | 10 trophozoites/mL | Not reported | (Haque, Kabir et al. 2010) |
| *Naegleria fowleri* | 18S rRNA | \| Forward: GTGCTGAAACCTAGCTATTGTAACTCAGT \| \| --- \| \| Reverse: CACTAGAAAAAGCAAACCTGAAAGG \| \| Probe: HEX-ATAGCAATATATTCAGGGGAGCTGGGC-BHQ \| | 153 | Taqman | 0.7 cells/PCR reaction | Not reported | (Qvarnstrom, Visvesvara et al. 2006) |
| Toxoplasma gondii | 35-fold repetitive B1 gene | \| Forward: TCCCCTCTGCTGGCGAAAAGT \| \| --- \| \| Reverse: AGCGTTCGTGGTCAACTATCGATTG \| \| Probe: FAM-TCTGTGCAACTTTGGTGTATTCGCAG-TAMRA \| | 98 | Taqman | 0.1 tachyzoites/PCR reaction | 90.6 | (Lin, Chen et al. 2000) |

**References**

Anuj, S. N., et al. (2009). "Identification of Pseudomonas aeruginosa by a duplex real-time polymerase chain reaction assay targeting the ecfX and the gyrB genes." Diagn Microbiol Infect Dis **63**(2): 127-131.

Behets, J., et al. (2007). "Development and evaluation of a Taqman duplex real-time PCR quantification method for reliable enumeration of Legionella pneumophila in water samples." J Microbiol Methods **68**(1): 137-144.

Blackstone, G. M., et al. (2007). "Use of a real time PCR assay for detection of the ctxA gene of Vibrio cholerae in an environmental survey of Mobile Bay." J Microbiol Methods **68**(2): 254-259.

Buckwalter, S. P., et al. (2012). "Real-time qualitative PCR for 57 human adenovirus types from multiple specimen sources." J Clin Microbiol **50**(3): 766-771.

Cashdollar, J. L., et al. (2013). "Development and evaluation of EPA method 1615 for detection of enterovirus and norovirus in water." Appl Environ Microbiol **79**(1): 215-223.

Clark, S. T., et al. (2011). "Evaluation of low-copy genetic targets for waterborne bacterial pathogen detection via qPCR." Water Res **45**(11): 3378-3388.

Costafreda, M. I., A. Bosch and R. M. Pinto (2006). "Development, evaluation, and standardization of a real-time TaqMan reverse transcription-PCR assay for quantification of hepatitis A virus in clinical and shellfish samples." Appl Environ Microbiol **72**(6): 3846-3855.

Fittipaldi, M., et al. (2011). "Discrimination of viable Acanthamoeba castellani trophozoites and cysts by propidium monoazide real-time polymerase chain reaction." J Eukaryot Microbiol **58**(4): 359-364.

Freeman, M. M., et al. (2008). "Enhancement of detection and quantification of rotavirus in stool using a modified real-time RT-PCR assay." J Med Virol **80**(8): 1489-1496.

Garcia-Quintanilla, A., et al. (2002). "Simultaneous identification of Mycobacterium genus and Mycobacterium tuberculosis complex in clinical samples by 5'-exonuclease fluorogenic PCR." J Clin Microbiol **40**(12): 4646-4651.

Guy, R. A., et al. (2003). "Real-time PCR for quantification of Giardia and Cryptosporidium in environmental water samples and sewage." Appl Environ Microbiol **69**(9): 5178-5185.

Haque, R., et al. (2010). "Diagnosis of amebic liver abscess and amebic colitis by detection of Entamoeba histolytica DNA in blood, urine, and saliva by a real-time PCR assay." J Clin Microbiol **48**(8): 2798-2801.

Jothikumar, N., et al. (2005). "Quantitative real-time PCR assays for detection of human adenoviruses and identification of serotypes 40 and 41." Appl Environ Microbiol **71**(6): 3131-3136.

Kageyama, T., et al. (2003). "Broadly reactive and highly sensitive assay for Norwalk-like viruses based on real-time quantitative reverse transcription-PCR." J Clin Microbiol **41**(4): 1548-1557.

Lambertz, S. T., et al. (2008). "Real-time PCR method for detection of pathogenic Yersinia enterocolitica in food." Appl Environ Microbiol **74**(19): 6060-6067.

Le Gall, F., et al. (2013). "Proposal of a quantitative PCR-based protocol for an optimal Pseudomonas aeruginosa detection in patients with cystic fibrosis." BMC Microbiol **13**: 143.

Li, B. and J. Q. Chen (2012). "Real-time PCR methodology for selective detection of viable Escherichia coli O157:H7 cells by targeting Z3276 as a genetic marker." Appl Environ Microbiol **78**(15): 5297-5304.

Lin, M. H., et al. (2000). "Real-time PCR for quantitative detection of Toxoplasma gondii." J Clin Microbiol **38**(11): 4121-4125.

Lin, W. S., C. M. Cheng and K. T. Van (2010). "A quantitative PCR assay for rapid detection of Shigella species in fresh produce." J Food Prot **73**(2): 221-233.

Lorente-Leal, V., et al. (2019). "Validation of a Real-Time PCR for the Detection of Mycobacterium tuberculosis Complex Members in Bovine Tissue Samples." Front Vet Sci **6**: 61.

Nogva, H. K., et al. (2000). "Application of the 5'-nuclease PCR assay in evaluation and development of methods for quantitative detection of Campylobacter jejuni." Appl Environ Microbiol **66**(9): 4029-4036.

Park, S. H., et al. (2011). "Multiplex PCR assay for the detection and quantification of Campylobacter spp., Escherichia coli O157:H7, and Salmonella serotypes in water samples." FEMS Microbiol Lett **316**(1): 7-15.

Qvarnstrom, Y., et al. (2006). "Multiplex real-time PCR assay for simultaneous detection of Acanthamoeba spp., Balamuthia mandrillaris, and Naegleria fowleri." J Clin Microbiol **44**(10): 3589-3595.

Radomski, N., et al. (2010). "Development of a real-time qPCR method for detection and enumeration of Mycobacterium spp. in surface water." Appl Environ Microbiol **76**(21): 7348-7351.

Rashid, R. B., et al. (2017). "Development and Validation of a Novel Real-time Assay for the Detection and Quantification of Vibrio cholerae." Front Public Health **5**: 109.

Sen, K., et al. (2011). "Development of a sensitive detection method for stressed E. coli O157:H7 in source and finished drinking water by culture-qPCR." Environ Sci Technol **45**(6): 2250-2256.

Verweij, J. J., et al. (2004). "Simultaneous detection of Entamoeba histolytica, Giardia lamblia, and Cryptosporidium parvum in fecal samples by using multiplex real-time PCR." J Clin Microbiol **42**(3): 1220-1223.

Verweij, J. J., et al. (2003). "Real-time PCR for the detection of Giardia lamblia." Mol Cell Probes **17**(5): 223-225.

Vezzulli, L., et al. (2015). "gbpA as a Novel qPCR Target for the Species-Specific Detection of Vibrio cholerae O1, O139, Non-O1/Non-O139 in Environmental, Stool, and Historical Continuous Plankton Recorder Samples." PLoS One **10**(4): e0123983.

Wang, H.-Y., et al. (2019). "Development of a high sensitivity TaqMan-based PCR assay for the specific detection of Mycobacterium tuberculosis complex in both pulmonary and extrapulmonary specimens." Scientific Reports **9**(1): 113.

Wilson, D. A., et al. (2003). "Detection of Legionella pneumophila by real-time PCR for the mip gene." J Clin Microbiol **41**(7): 3327-3330.

Wong, S. Y., et al. (2014). "Insertion/deletion-based approach for the detection of Escherichia coli O157:H7 in freshwater environments." Environ Sci Technol **48**(19): 11462-11470.

Yanez, M. A., et al. (2005). "Quantitative detection of Legionella pneumophila in water samples by immunomagnetic purification and real-time PCR amplification of the dotA gene." Appl Environ Microbiol **71**(7): 3433-3441.

Yang, C., et al. (2003). "Application of real-time PCR for quantitative detection of Campylobacter jejuni in poultry, milk and environmental water." FEMS Immunol Med Microbiol **38**(3): 265-271.

Yang, G., et al. (2010). "Dual detection of Legionella pneumophila and Legionella species by real-time PCR targeting the 23S-5S rRNA gene spacer region." Clin Microbiol Infect **16**(3): 255-261.
