# Supplementary material for: Wastewater-based surveillance of microbial pathogens in GCC countries (2015–2025): a scoping review and questionnaire survey with stakeholders
Source: Front Public Health. 2026 Apr 22;14:1786753. doi: 10.3389/fpubh.2026.1786753 (PMC13144031; doi:10.3389/fpubh.2026.1786753)
Supplement: Supplementary file 4 [file Table_4.docx]

**Supplementary Material 1**

Boolean query for literature collection

A structured literature search was conducted across four electronic databases: Web of Science, Scopus, PubMed, and Google Scholar. The search aimed to identify peer-reviewed articles published in English between January 2015 and October 2025 related to wastewater surveillance in the Gulf Cooperation Council (GCC) region.

The following exact Boolean query was applied across the search fields (e.g., Title/Abstract/Keywords) in all four databases:

("wastewater-based surveillance" OR "wastewater monitoring" OR "sewage monitoring" OR "wastewater-based epidemiology") AND ("Gulf Cooperation Council" OR "GCC" OR "Saudi Arabia" OR "United Arab Emirates" OR "UAE" OR "Kuwait" OR "Qatar" OR "Oman" OR "Bahrain")
