# Supplementary material for: Wastewater-based surveillance of microbial pathogens in GCC countries (2015–2025): a scoping review and questionnaire survey with stakeholders
Source: Front Public Health. 2026 Apr 22;14:1786753. doi: 10.3389/fpubh.2026.1786753 (PMC13144031; doi:10.3389/fpubh.2026.1786753)
Supplement: Supplementary file 6 [file Data_Sheet_2.pdf]

Gulf CDC Survey on Wastewater-Based Surveillance (WBS) in GCC Countries

This survey aims to assess the current state of wastewater-based surveillance (WBS) across GCC countries by:

1. **Mapping core capabilities** for detecting communicable diseases and antimicrobial-resistance (AMR) signals and patterns.
2. **Evaluating monitoring tools** used to identify illicit substance use in sewer catchments.
3. **Assessing analytical capacity** for tracking chemical contaminants and broader environmental hazards.
4. **Identifying gaps and priorities** in infrastructure, laboratory methods, and data-sharing mechanisms.

Findings will guide regional coordination efforts led by the Gulf CDC and build on the WHO/UN Wastewater Surveillance (WES) guidance (Dec 2024).

Background Information

1. What is your Country *

- Bahrain
- Kuwait
- Oman
- Qatar
- Saudi Arabia
- United Arab Emirates

2. Institution Name *

3. Type of Organization *

- ◯ Ministry of Health
- ◯ Public Health Institute
- ◯ Environmental Agency
- ◯ Water/Sanitation Utility
- ◯ Academic/Research Institution
- ◯ Laboratory
- ◯ Other

4. Your Role/Title *

5. Name *

6. E-mail *

Current WBS Activities

7. Do you have action-plans, strategies, policies, manuals for conducting wastewater surveillance? *

- ○ Yes
- ○ No
- ○ Not sure

8. Is your organization currently involved in WBS? *

- ○ Yes
- ○ No
- ○ Planning to start soon

9. If planning to start, what is the expected date *

[ ]

10. Which pathogens are currently targeted? *(check all that apply)* *

- ☐ SARS-CoV-2
- ☐ Polio
- ☐ Cholera
- ☐ Typhoid/Paratyphoid
- ☐ Antimicrobial resistance (AMR) markers
- ☐ Influenza A/B
- ☐ Monkeypox
- ☐ Noroviruses
- ☐ Bacterial pathogens
- ☐ Other

11. What is the main purpose of your WBS? *

- ◯ Early warning
- ◯ Trend monitoring
- ◯ Genomic surveillance
- ◯ Supplement to case-based systems
- ◯ Research only
- ◯ Other

12. Where do you perform your WBS? (Check all that apply) *

- ☐ Manholes
- ☐ Wastewater treatment plants
- ☐ Aircraft lavatories
- ☐ Hospitals
- ☐ Other

Pathogen Prioritization and Public Health Relevance

13. Which pathogens have you prioritized for WBS in your context? (Rank top 5) *

14. Why are these a priority? *(check all that apply)* *

- ☐ High disease burden
- ☐ Risk of outbreak or spread
- ☐ Gaps in clinical surveillance
- ☐ Public health urgency
- ☐ Regional relevance
- ☐ Vaccine-preventable
- ☐ Other

Feasibility and Infrastructure

15. What is the level of sewerage coverage in the areas you perform WBS with? *

- ◯ Mostly sewered
- ◯ Mostly non-sewered
- ◯ Mixed
- ◯ Not sure

16. What types of sampling sites are used or considered? (c*heck all that apply*) *

- ☐ Wastewater treatment plants (WWTPs)
- ☐ Sewer manholes
- ☐ Hospitals/institutions
- ☐ Ports/airports
- ☐ Non-sewered areas
- ☐ Other

17. Which lab capacities / methods are available? *(check all that apply)* *

- ☐ Sample concentration
- ☐ PCR/qPCR
- ☐ Next generation sequencing
- ☐ Cultivation
- ☐ Antimicrobial resistance analysis
- ☐ None
- ☐ Outsourced

18. Do environmental conditions affect WBS? *(check all that apply)* *

- Sample degradation
- Low/no water flow
- Difficult sampling
- No major impact
- Maybe

19. Is treated wastewater reused in your country ? *

- Yes – extensively
- Yes – limited
- No
- Maybe

Type and Future of WBS Activities

20. Which types of WBS activities are you currently engaged in? *

- ◯ Routine/sentinel surveillance
- ◯ Agile/outbreak-focused surveillance
- ◯ Planning/preparedness only
- ◯ Not involved
- ◯ Other

21. Which components would you like to scale up? (*check all that apply*) *

- ☐ Routine surveillance
- ☐ Emergency response
- ☐ System development
- ☐ Not sure

22. What are the main challenges to scale-up? *(check all that apply)* *

- ☐ Funding
- ☐ Technical capacity
- ☐ Trained workforce
- ☐ Data systems
- ☐ Governance/legal
- ☐ Infrastructure
- ☐ Other

Data to Action

23. How is WBS data analyzed and interpreted? *

- ◯ Internally
- ◯ By public health agency
- ◯ Outsourced
- ◯ Not yet analyzed
- ◯ Other

24. Who receives WBS data? *

- ◯ National public health
- ◯ Local health units
- ◯ Environmental regulators
- ◯ Emergency response teams
- ◯ Not used
- ◯ Other

25. Who uses WBS data? *

- ◯ National public health
- ◯ Local health units
- ◯ Environmental regulators
- ◯ Emergency response teams
- ◯ Not used

26. Is WBS data used to inform public health action? *

- ◯ Yes – routinely
- ◯ Sometimes
- ◯ Rarely
- ◯ Not at all
- ◯ Maybe

27. Barriers to translating WBS into action? *(check all that apply)* *

- ☐ Poor integration and forecasting abilities
- ☐ Data limitations
- ☐ No standard protocol to follow
- ☐ Legal/political constraints
- ☐ No clear response framework
- ☐ Low awareness
- ☐ Other

28. What would improve WBS impact? *(check all that apply)* *

- ☐ Clear protocols
- ☐ Real-time or forecasting data tools
- ☐ Training
- ☐ Policy/legal support
- ☐ Collaboration and coordination
- ☐ Other

Multisector Coordination

29. Which sectors are involved in WBS in your country? *

- ◯ Health
- ◯ Environment
- ◯ Sanitation
- ◯ Academic
- ◯ National lab network
- ◯ Municipal authorities
- ◯ Other

30. Is there a formal coordination mechanism? *

- ◯ Yes – national
- ◯ Yes – informal/working group
- ◯ No
- ◯ Maybe

31. How is data shared between sectors? *

- ◯ Joint reports
- ◯ Informal comms
- ◯ Shared data platforms
- ◯ No routine sharing
- ◯ Other

32. Challenges in cross-sector collaboration? *(check all that apply)* *

- [ ] Siloed mandates
- [ ] No clear lead
- [ ] Data/privacy issues
- [ ] Competing priorities
- [ ] Resource constraints
- [ ] Other

Regional & Cross-Border Relevance

33. Do you consider cross-border risks (e.g., Hajj, migrant labor, refugees)? *

- ◯ Yes – routinely
- ◯ Occasionally
- ◯ No
- ◯ Not applicable

34. Would you support a regional WBS initiative (e.g., for mass gatherings)? *

- ◯ Yes
- ◯ Maybe
- ◯ No

Ethical, Legal, and Social Acceptability

35. Have any ethical concerns been raised about WBS in your setting? *

- ◯ Yes – from communities
- ◯ Yes – from institutions and/or decision-makers
- ◯ No concerns so far
- ◯ Maybe

36. Are there privacy or stigma-related risks associated with your WBS activities? *

- ◯ Yes – e.g., risk of community targeting or misinterpretation
- ◯ No known risks
- ◯ Not assessed
- ◯ Not applicable (large-scale, anonymized sampling only)

37. Are there any legal or regulatory barriers to conducting WBS? *

- ◯ Yes – sample ownership/legal authorization issues
- ◯ Yes – unclear policies around data use or ethics
- ◯ No known legal barriers
- ◯ Maybe

38. Is there a formal ethics or legal review process for your WBS activities? *

- ◯ Yes – all activities are reviewed
- ◯ Some activities are reviewed
- ◯ No formal process
- ◯ Maybe

Capacity Building and Support Needs

39. What support would benefit your WBS program? *(check all that apply)* *

- ☐ Training
- ☐ Equipment
- ☐ Technical guidance
- ☐ Policy support
- ☐ Funding
- ☐ Stakeholder coordination
- ☐ Data/IT tools
- ☐ Regional collaboration
- ☐ Other

40. Are you interested in joining a regional WBS network coordinated by Gulf CDC? *

- ◯ Yes
- ◯ Maybe
- ◯ No

41. If the sewage system does not adequately represent the risk for the entire country (or targeted area), would you still consider using it for surveillance? Alternatively, would you prefer to use random sampling? *

42. Do you have any additional suggestions or comments on strengthening WBS in your country or the GCC?

This content is neither created nor endorsed by Microsoft. The data you submit will be sent to the form owner.

Microsoft Forms
